# Methodology for ESR iGuide content

**DOI:** 10.1186/s13244-019-0720-z

**Published:** 2019-03-13

**Authors:** European Society of Radiology (ESR)

**Affiliations:** Vienna, Austria

**Keywords:** Imaging referral guidelines, Clinical decision support, Appropriate imaging, Radiation protection, Patient safety

## Abstract

The European Society of Radiology (ESR) considers the use of evidence-based referral guidelines in clinical practice essential to ensure the appropriate utilisation of medical imaging for patients. Since 2014, the ESR has been working with the American College of Radiology (ACR) to develop referral guidelines for Europe, based on the ACR Appropriateness Criteria (AC) and appropriate use criteria developed by the ACR Rapid Response Committee.

This paper sets out the methodology used by the ESR’s Referral Guidelines Subcommittee to adapt the ACR criteria for use in the European clinical decision support (CDS) platform ESR iGuide. The ESR adheres to the ACR’s original methodology as far as applicable, and has established additional methodological guidance for its experts, establishing several key principles:

- Any changes to existing recommendations, and any additional guidelines, should be based on evidence as far as possible
- Expert opinion, judgement, European practice standards, should only function as a supplement when necessary
- Appropriateness recommendations should give no consideration to national or institutional circumstances, costs, or availability of equipment.

The cooperation between the ACR and ESR on appropriate use criteria and imaging referral guidelines provides added value to both societies as European studies and guidelines from European subspecialty societies of radiology are fed into the process and the exchange of views among the experts makes the guideline development and review process more robust.

The ESR’s aim is to ensure referrers and patients benefit from the best possible guidance for appropriate imaging.

## Key points

- ESR develops guidelines designed for use in a decision support software
- The ESR bases their recommendations on a review and adaptation of the ACR Appropriateness Criteria
- Recommendations are based on the best-available evidence supplemented where necessary with expert opinion and practice standards
- Guidelines are periodically released in coordination between ACR and ESR

## Introduction

The European Society of Radiology (ESR) considers referral guidelines for medical imaging essential for improving the appropriateness and justification of radiological procedures. To address gaps in the availability of imaging referral guidelines and the lack of guideline use in Europe, the ESR decided to develop a clinical decision support system for European imaging referral guidelines. The most efficient way to introduce guidelines for use in a clinical decision support system at the European level is to leverage existing efforts. Having assessed several options, the ESR entered into a cooperation with the American College of Radiology (ACR) in 2014. Their Appropriateness Criteria (AC) were already in use in the decision support system ACR Select, and following an open call for proposals the ESR selected the National Decision Support Company (NDSC) platform, also used for ACR Select, to deliver the ESR’s guidelines.

The ESR initially appointed a project group for a rapid review process of the ACR Select content. The content was divided into nine topic areas, and nine radiologists selected by the ESR reviewed and adapted the CDS guidelines for use in ESR iGuide within one year.

This group was transformed into the permanent ESR Imaging Referral Guidelines Working Group (IRG WG) in 2016, subsequently turned into the ESR Referral Guidelines Subcommittee (RGSC) in 2018, as part of the ESR’s Quality, Safety and Standards Committee (QSSC). The purpose of this RGSC is to maintain the content of ESR iGuide and review and adapt future versions of the ACR Select content for use in Europe.

This document sets out the process and methodology according to which the ESR RGSC reviews and modifies the ACR Select content.

## Methodology

### General considerations

The ACR Select content is composed of the ACR AC for diagnostic imaging, which are evidence-based and developed according to a widely accepted, transparent methodology [1–4]. The ACR AC satisfy federal appropriate use criteria (AUC) in the United States, which is a prerequisite for the ACR’s status as a qualified Provider-Led Entity (qPLE) of the Centers for Medicare & Medicaid Services (CMS) under the Medicare Appropriate Use Criteria programme. This means that medical providers can consult ACR Appropriateness Criteria to fulfil impending Protecting Access to Medicare Act (PAMA) requirements to consult AUC prior to ordering advanced diagnostic imaging for Medicare patients.

The ACR Select content includes additional guidelines developed by the ACR’s Rapid Response Committee (RRC), created using the evidence base of the ACR AC supplemented by expert consensus and opinion, and the experience of using CDS in clinical practice. The purpose of this additional content is to widen the coverage of the guidelines to areas where the strength of evidence may be insufficient to develop an ACR AC topic.

The methodology in use for the ACR AC has evolved from the work of the Rand Corporation in the late 1980s. Numerous specialty societies have adopted and used the general approach, including the methodology of the ACR.

The work of the ESR’s RGSC of reviewing and adapting the ACR’s clinical imaging guidelines (CIG) builds upon the methodology used for the development of the ACR Appropriateness Criteria, which is in line with the RAND method. The ESR’s methodology’s aim is to ensure that the review and adaptation process is evidence-based to the largest extent possible. Appropriateness ratings reflect medical necessity and the diagnostic value of diagnostic imaging studies.

Inherent in guidelines is the focus on balancing the possible benefit against the possible risk. It is clear that there are at the very least potential negative consequences of radiation; the concerns are greater in younger patients, due to the latency of these potential adverse effects. The cost of the imaging exam is another important variable. Depending on the nature of the healthcare system, this may not be a concern to the individual undergoing the imaging exam, but it is always a concern to the system as a whole. If the likelihood is very low or negligible that a specific imaging exam in a specific clinical setting is going to provide useful information - for example a routine chest radiograph in an otherwise healthy young adult non-smoker - then even a modest cost is hard to justify. For most guidelines, both cost and radiation, then, are always part of the risk-benefit equation. The aim of CIG is to provide the best possible advice in specific clinical settings, realising that with the clear limitations in knowledge, the many specific clinical variables (age, gender, medical history, environmental and familial risk factors), and available expertise and equipment, a definitive recommendation may not always be possible.

In the European setting, the ESR’s guidelines should be equally valid across a variety of different health systems. Therefore, while radiation risk is one of the variables to consider in the justification of an imaging study in a given clinical situation, the consideration of the cost of these studies must necessarily be disregarded in the ESR content version. The resulting guidelines should be generic to be widely applicable, and can be localised to account for specific variables such as cost and availability when guidelines are implemented in a particular setting.

There are also reasons other than medical necessity to consider imaging. These include patient preference; a patient may want a CT scan for back pain, simply for personal reassurance. Also, many healthcare providers feel that they can both reassure themselves and their patients and perhaps lessen the risk of a malpractice accusation if an imaging exam is requested even if there is no real concern for negligence. There may also be financial incentives to getting an imaging study, if the referring provider has a fiduciary interest in the imaging equipment. It may simply be expeditious: it can be faster to get an imaging study than to carefully evaluate a patient in a busy emergency room, or explain at length to an anxious patient that an imaging study is unlikely to be clinically useful. The use of CIG is an effective means to deal with many of these non-medical reasons for obtaining imaging. Again, the entry point for CIG is the question: which, *if any*, imaging exam is most likely to be helpful in the diagnosis and care of this patient in this clinical setting. Therefore, the diagnostic value, in relation to the potential radiation risk, should be the deciding factor for each appropriateness rating.

### Composition of the ESR RGSC

The membership of the ESR RGSC is determined in accordance with the ESR’s statutes. The chair of the RGSC proposes members in consultation with the chair of the QSSC. All nominees must be approved by the ESR Executive Council.

As of 2019, the membership of the RGSC consists of experts for each of the 10 topic areas in ESR iGuide, and members’ expertise (subspecialty training) must correspond to these topic areas. For each topic area, the expert(s) are representatives from the appropriate European subspecialty society of radiology and/or chosen from the general ESR membership.

Topic areas:

- Breast Imaging
- Cardiac Imaging
- Gastrointestinal Imaging
- Musculoskeletal Imaging
- Neurologic Imaging
- Paediatric Imaging
- Thoracic Imaging
- Urologic Imaging
- Vascular Imaging
- Women’s Imaging

Each of the appointed experts reviews and modifies the guidelines in his assigned topic area, conducts literature and evidence reviews and consults colleagues and other specialists at his discretion.

### Principles of the ESR guideline review

The methodology for the ACR Appropriateness Criteria is the basis for the work of the ESR RGSC and is adhered to as far as applicable. The basic principles are that any changes ESR experts make to the guidelines should be based on scientific evidence to the largest extent possible. Expert opinion, judgement and experience of European practice standards can function as a supplement where necessary. No consideration should be given to national, local, or institutional circumstances such as national regulations, cost or reimbursement rates, or the availability or quality of imaging equipment, as the resulting guidelines should be generic and equally applicable anywhere in Europe. To account for local circumstances, the ESR iGuide can be modified for that particular situation during implementation.

The guidelines contain over 1,600 indications (March 2018) for which different radiological examinations have been evaluated in terms of their diagnostic value and appropriateness. The ESR also applies the most widely used rating scale from 1 to 9, with 1–3 defined as “not usually appropriate”, 4–6 defined as “may or may not be appropriate” and 7–9 defined as “usually appropriate” according to the ACR methodology. Each indication is applicable for defined patient groups (age range: 0–150 years; male/female/both). Like the ACR, the ESR follows the RAND/UCLA Appropriateness Method User’s Manual [5] where “the expected health benefit (e.g., increased life expectancy, relief of pain, reduction in anxiety, improved functional capacity) exceeds the expected negative consequences (e.g., mortality, morbidity, anxiety, pain, time lost from work) by a sufficiently wide margin that the procedure is worth doing, exclusive of cost”.

**Table:** 

Appropriateness Category Name	Appropriateness Rating	Appropriateness Category Definition
Usually Appropriate	7, 8, or 9	The imaging procedure or treatment is indicated in the specified clinical scenarios at a favourable risk-benefit ratio for patients.
May Be Appropriate	4, 5, or 6	The imaging procedure or treatment may be indicated in the specified clinical scenarios as an alternative to imaging procedures or treatments with a more favourable risk-benefit ratio, or the risk-benefit ratio for patients is equivocal.
Usually Not Appropriate	1, 2, or 3	The imaging procedure or treatment is unlikely to be indicated in the specified clinical scenarios, or the risk-benefit ratio for patients is likely to be unfavourable.

The content is divided into 10 radiological subspecialty or topic areas,^1^ each reviewed by dedicated experts nominated by the ESR (see 2) b.). Each expert’s task is to review all indications, exams and appropriateness ratings within his designated area to either confirm agreement with the appropriateness rating of the ACR, or to change it by citing applicable literature not considered as part of the ACR evidence. The cited literature will typically be European guidelines for imaging, European-based studies or recently published literature that was not yet available at the time that the ACR AC guidelines were developed. Additional changes may be made based on expert consensus or opinion in the absence of sufficient literature evidence provided a justification can be given.

As part of the review process, ESR experts are expected to survey the ACR evidence. Based on the strength of evidence and assessment by the ACR for each AC topic, provided in evidence tables with information on the study type, number of patients, study objective, study results, and an assessment of the studies quality, reviewing experts consider additional literature not yet covered in the ACR AC development process when determining whether to change an appropriateness rating. This literature should be high quality, peer-reviewed, and publicly available, and can include guidelines issued by European subspecialty societies of radiology. Appropriateness ratings for the European edition of the guidelines are changed if the evidence is considered sufficient, and/or if expert consensus and/or opinion are sufficiently strong to make a guideline compatible with European standards of practice.

For all changes, an explanatory comment is added to provide a reason for changing the rating. The ESR has kept the RGSC deliberately small to ensure an efficient process, but ESR experts are encouraged to work with or consult colleagues during the review at their discretion to solicit different opinions and discuss changes.

### Review and adaptation process

The experts review each indication falling into their area of expertise. A number of scored (i.e. rated based on appropriateness) imaging exams are associated with each examination.

The review and adaptation process is as follows:

1. Review of indication name
2. Review exam names associated with the indications
3. Review of ACR documentation: narrative, rating table, evidence table, referenced literature
4. Determination if indication name is adequate for Europe
  if not: proposal for changing indication name
  Indication names are based on ACR Commons terminology or ICD, so changes to indication names should only be made if an indication is unclear or confusing
5. Determination whether the associated exams are relevant for the clinical indication

- if not: changing, removing, or adding exams
- Exam names and modifiers should follow the ACR Commons terminology

6. Review of the appropriateness rating of exam for every indication based on ACR evidence

- Agreement with appropriateness rating: no further action
- Change in appropriateness rating: review of additional literature; citation of literature to justify appropriateness change; references to be added to review sheet
  - Evidence needs to be strongest for changes to ACR AC topics and for changes across appropriateness category (as opposed to rating changes within a category)
  - Literature search: assessment of available additional studies, guidelines, other relevant publications
  - Revision of appropriateness rating to reflect the additional evidence found
  - Citations are edited to add the literature considered by the ESR
  - In addition: expert comment to explain the change
  - In the absence of sufficient literature evidence, all changes to appropriateness ratings are marked as “Changed based on ESR expert opinion”; preferably, this should only be done to non-ACR AC topics and should only be rating changes within the same category

7. Review of the applicable patient characteristics
  age range: 0–150 years
  sex: male, female, both/unknown

To summarise, the content can be adapted by ESR experts in the following ways:

- Changes to appropriateness ratings: referenced literature evidence and explanatory comments; at expert discretion only in exceptional cases
- Changes to indication names: based on evidence or at expert discretion
- Changes to exam names: based on evidence or at expert discretion
- Changes to age range for which a guideline applies for: based on evidence or at expert discretion
- Changes to sex for which a guideline applies for: based on evidence or at expert discretion
- New rules (indications plus rated exams): based on referenced literature evidence and explanatory comments

In addition, the entirety of the content within a topic area should be reviewed to ensure:

- relevance of all indications/exams (according to ACR methodology for topic selection)
- avoidance of unnecessary overlap/duplication between indications (esp. between ACR AC topics and additional RRC/ESR content)

All ESR content changes are communicated to the ACR RRC for consideration and discussed in periodic meetings between the RGSC and RRC. Furthermore, ESR changes are communicated to the ACR panels for consideration in future revisions of the ACR Appropriateness Criteria.

## ESR guideline releases, updates, future process

The ESR releases new editions of the ESR iGuide content approximately once a year. The first two content versions included changes to the appropriateness category of the reviewed imaging studies in less than 10% of cases, indicating a very high degree of consensus between the ACR and ESR expert groups.

The ESR reviews new versions of the ACR Select content after they are released in the United States. All modifications, additions and deletions are documented so that ESR experts understand exactly what has changed since the previous edition.

In the future, the ESR and ACR plan to have the ACR RRC and the ESR RGSC working simultaneously on new CDS content versions, and releasing two editions with different ACR and ESR appropriateness ratings at the same time.

## Footnotes

1 A 10th topic area, paediatric imaging, was added to the ACR Select and ESR iGuide content in 2017.
